# Antimicrobial effects of novel *Hermetia illucens* peptides

**DOI:** 10.1038/s41598-026-40997-3

**Published:** 2026-02-24

**Authors:** Emine Derin, Laurence Van Moll, Milan Wouters, Linda De Vooght, Federica De Stefano, Carmen Scieuzo, Paul Cos, Patrizia Falabella

**Affiliations:** 1 Department of Basic and Applied Sciences, University of Basilicata, Potenza, Italy; 2 Laboratory for Microbiology, Parasitology and Hygiene (LMPH), Faculty of Pharmaceutical, Biomedical and Veterinary Sciences, University of Antwerp, Antwerp, Belgium; 3 Spinoff XFlies s.r.l., University of Basilicata, Via dell’Ateneo Lucano 10, Potenza, 85100 Italy

**Keywords:** Antimicrobial peptides, Black Soldier Fly, Defensin, Antibacterial activity, Biotechnology, Drug discovery, Microbiology

## Abstract

Antimicrobial resistance represents a significant global health challenge, implicated in nearly 5 million deaths per year. This study investigates the antimicrobial properties of selected peptides derived from the Black Soldier Fly (BSF), identified *in silico*. Ten synthesized peptides were evaluated *in vitro* against Gram-positive bacteria (*Staphylococcus aureus*, *Bacillus cereus*), Gram-negative bacteria (*Escherichia coli*, *Pseudomonas aeruginosa*), and fungi (*Aspergillus fumigatus*, *Candida albicans* ). Cytotoxicity was assessed using human lung fibroblasts and red blood cells. Among the peptides, Hill_BB_C7176 demonstrated broad-spectrum antibacterial activity, no cytotoxicity and low hemolytic activity (IC₅₀: 31.6 - >32 µM). The peptide displayed rapid bactericidal activity achieving complete eradication at 8× its minimal inhibitory concentration. Propidium iodide uptake increased in a concentration dependent manner, indicating membrane permeabilization contributing to antibacterial activity. In addition, Hill_BB_C7176 exhibited strong binding affinity to lipopolysaccharide (lipid A). *In vivo*, Hill_BB_C7176 improved survival of *Galleria mellonella* larvae infected with *S. aureus* and *E. coli*, showing similar survival outcomes to reference antibiotics under the experimental conditions tested. These findings highlight the antibacterial potential of BSF-derived antimicrobial peptides, particularly Hill_BB_C7176, supporting the further research on the antibacterial activity of BSF antimicrobial peptides.

**Supplementary Information:**

The online version contains supplementary material available at 10.1038/s41598-026-40997-3.

## Introduction

 The global rise of antimicrobial resistance (AMR) is a significant threat to the effectiveness of existing antibiotics and presents a critical challenge to modern medicine. In 2019, AMR was responsible for approximately 4.9 million deaths worldwide, primarily due to *Escherichia coli *and *Staphylococcus aureus*^1,2^. Until new therapies are discovered, AMR is expected to become a major cause of death by 2050^2,3^. Therefore, identifying antimicrobial agents with novel mechanisms of action is an urgent global priority.

In this context, antimicrobial peptides (AMPs), which are short, naturally occurring, gene-encoded molecules, have become promising alternatives to conventional antibiotics. AMPs often display rapid bactericidal activity against diverse microbial species, while generally exhibiting a lower propensity for resistance development compared to classical antibiotics^4,5^. AMPs can be effective against a wide variety of bacteria, as well as viruses, fungi, and parasites. AMPs usually target and disrupt the cell membranes of Gram-negative and Gram-positive bacteria, thereby exhibiting a direct bactericidal effect. Their rapid action and structural versatility make them attractive candidates for next-generation antimicrobials^6–8^.

All living organisms naturally produce AMPs as an essential component of their innate immune defense against infections^9–11^. Insects represent a particularly rich and underexplored reservoir of AMPs due to their adaptation to microbe-rich environments^12^. The Black Soldier Fly (BSF, *Hermetia illucens*) has emerged as a promising AMP source, producing peptides such as defensins, cecropins, and attacins with notable antibacterial and antifungal activity^8,13–17^.

This study presents an exploratory analysis of the antimicrobial activity and cytotoxicity of a novel library of BSF-derived AMPs. The peptides were previously selected among 57 AMP sequences, which were analyzed using *in silico* bioinformatics methods^18^. Selection was based on computational predictions using CAMPR3 machine learning algorithms, followed by filtering for optimal physicochemical properties, including clustering score (> 0.7), net charge (0 to + 8), and molecular weight (3000–5500 Da). Peptides meeting these criteria were prioritized due to their favorable predicted activity and structural characteristics, providing a strong foundation for subsequent experimental validation^19,20^. By performing a first, exploratory characterisation of new BSF AMPs, this study aims to expand the knowledge base for the development of next-generation antimicrobial therapies. Given the urgent need for novel strategies to address AMR, the exploration of BSF-derived AMPs represents an important step forward in antimicrobial drug discovery.

## Materials and methods

### Sample preparation

AMP sequences derived from a *Hermetia illucens* transcriptome, previously published and analysed in Moretta *et al.*, 2020^18^. The putative AMPs that showed high antimicrobial score values with all prediction software were selected and chemically synthesised (solid-phase synthesis/Fmoc chemistry) (Bio-Fab Research, Rome, Italy)^18,21^. The peptide characteristics, including type, amino acid sequence and length, purity, water solubility, molecular weight, total hydrophobic ratio, isoelectric point, are detailed in Supplementary Table S1. Peptides were initially dissolved in dimethyl sulfoxide (DMSO; Acros Organics) and double-distilled water (ddH_2_O) at a concentration of 5 mM, followed by further dilution in sterile demineralized water. Purity (> 95% for all peptides) was determined via high-performance liquid chromatography and mass spectrometry by the peptide producer^16^.

### Cytotoxicity screening of MRC5-SV2 cells

To assess early signs of peptide-induced cytotoxicity, the peptide library was tested against the MRC5-SV2 human embryonic lung fibroblast cell line (Millipore, Burlington, MA, USA). Peptides were serially diluted in 96-well plates at concentrations ranging from 32 µM to 0.25 µM for duplicate screenings. Tamoxifen (Sigma-Aldrich), was selected as a reference compound due to its well-documented cytotoxicity in normal human cell lines, providing a reliable benchmark for comparison^16,22,23,24^. Tamoxifen, following in-house optimized protocols, was tested in a ½ serial dilution range from 64 µM to 0.5 µM. A 190 µL cell suspension containing 1.5 × 10^5 cells/mL in complete minimum essential medium (Gibco) was added to the peptide-containing wells. Plates were incubated at 37 °C in a 5% CO_2_ atmosphere for 72 h. After incubation, 50 µL of a 0.01% (w/v) resazurin solution (Sigma-Aldrich) was added to each well, and fluorescence was measured using a microplate reader (Infinite F Plex, Tecan) at λexcitation = 550 (10) nm and λemission = 590 (10) nm after 4 h of incubation. IC₅₀ values were determined. 

### Hemolysis analysis for the assessment of cytotoxicity

Hemolysis analysis was performed using fresh human whole blood (5–6 mL) collected in tubes containing 30 units of heparin (approved by the Ethics Committee of the University of Antwerp, approval number EDGE 004493)^25^. The blood was centrifuged at 1,000 x g for 5 min and repeatedly washed with 1X phosphate-buffered saline (PBS; Gibco) until the supernatant became clear. The erythrocyte pellet was then resuspended in 1X PBS to obtain a 2% red blood cell suspension. Serial dilutions of AMPs (150 µL) in 1X PBS were mixed with 150 µL of the red blood cell suspension in microcentrifuge tubes. As controls, 0.1% (v/v) Triton X-100 (Sigma-Aldrich) (positive control) and 1X PBS (negative control) were included. Samples were incubated at 37 °C for 1 h, followed by centrifugation at 1,000 x g for 5 min. Subsequently, 200 µL of the supernatant was transferred to a 96-well plate, and absorbance was measured using a microplate reader (Infinite F Plex, Tecan) at an excitation wavelength of 550 (10) nm and an emission wavelength of 590 (10) nm to assess hemoglobin release. Each assay was conducted in biological duplicate. The hemolysis percentage was calculated as [(sample absorbance − negative control absorbance)/(positive control absorbance − negative control absorbance)] × 100. IC₅₀ values were determined^16,24,26–28^.

### Bacterial isolates and culture conditions

The bacterial strains used in this study included *Staphylococcus aureus *ATCC 6538, *Escherichia coli* ATCC 8739, *Pseudomonas aeruginosa* ATCC 9027, and *Bacillus cereus *ATCC 14,579, obtained from the American Type Culture Collection (ATCC, Manassas, VA, USA). *Aspergillus fumigatus* B42928 and *Candida albicans* B59630 were acquired from the Belgian Coordinated Collections of Microorganisms. Bacteria were cultured in Mueller-Hinton broth (MHB; Difco) and on Mueller-Hinton agar (MHA; Sigma-Aldrich) or tryptic soy agar (TSA; Sigma-Aldrich), while fungal species were grown in Roswell Park Memorial Institute (RPMI; Gibco) medium.

### Antimicrobial screening assay

Peptides were serially diluted in 96-well plates using an automated liquid-handling workstation (Beckman Coulter Biomek 3000) with a final volume of 10 µL per well. The final in-plate concentration of DMSO did not exceed 1%. Peptide concentrations ranged from 32 µM to 0.016 µM, and each assay was performed in duplicate. Reference compounds included doxycycline (Sigma-Aldrich) for bacterial strains (*S. aureus*, *E. coli*, *P. aeruginosa*, and *B. cereus*), flucytosine (Sigma-Aldrich) for *C. albicans*, and econazole (Sigma-Aldrich) for *A. fumigatus*. Standard reference antimicrobials, including doxycycline (antibacterial), flucytosine, and econazole (antifungal), were used as positive controls at concentrations ranging from 0.016 to 8 µM. A bacterial/fungal suspension (5 × 10^3^ CFU/mL for *S. aureus*, *E. coli*, *B. cereus*, *A. fumigatus* and *C. albicans*, and 5 × 10^4^ CFU/mL for *P. aeruginosa*) was prepared in MHB or RPMI and added to the 96-well plates. Incubation was conducted at 37 °C for 16 h (*S. aureus*, *E. coli*, *P. aeruginosa*, and *B. cereus*), 24 h (*C. albicans*), or 48 h (*A. fumigatus*). Antimicrobial activity was assessed using a resazurin assay. Fluorescence was measured using a microplate reader (Infinite F Plex, Tecan) at λexcitation = 550 (10) nm and λemission = 590 (10) nm. Concentrations leading to 50% growth inhibition of the pathogen (IC₅₀ ) and Minimum Inhibitory Concentration (MIC) values were determined with IC₅₀ calculated using linear interpolation and MIC assessed visually. Minimum Bactericidal Concentrations (MBC) were established by plating samples on MHA, where MBC was defined as the lowest peptide concentration that established a minimum of a 3-log reduction in viable bacteria compared to the growth controls^16,29^.

### Time-kill analysis

The antibacterial kinetics of the selected AMP Hill_BB_C7176 were analyzed following Mascio *et al.*^30^ Each strain was cultured to its mid-logarithmic phase, corresponding to an optical density OD₆₀₀ ≈ 0.4–0.6 for *S. aureus* and 0.5–0.8 for *E. coli*. Bacterial suspensions were adjusted to approximately 1 × 10⁸ CFU/mL in Mueller–Hinton broth. Serial peptide dilutions (10 µL) were mixed with 190 µL in 96-well plates and incubated at 37 °C. At predetermined time points (0, 0.5, 1, 2, and 4 h), aliquots were serially diluted in 1X PBS and spot plated on TSA by spotting 10 µL droplets of each sample. After overnight incubation at 37 °C, colonies were counted. Bactericidal activity was defined as a ≥ 3-log10 reduction in bacterial count compared to the control. Time points of 0, 0.5, 1, 2, and 4 h were selected to evaluate both rapid and sustained bactericidal effects based on preliminary results suggesting early peptide activity. Bacterial counts were averaged from three independent experiments and visualized using GraphPad Prism 8^16,24,30^.

### Inner membrane permeabilization by the propidium iodide uptake assay

To assess inner membrane damage, a propidium iodide (PI; Thermo Fisher) uptake assay was conducted as per Dassanayake *et al.*^31^. *S. aureus* and *E. coli* cultures were grown to mid-log phase in MHB, centrifuged at 3,000 x g for 15 min, and resuspended in 5 mM HEPES (Sigma-Aldrich) buffer (pH 7.4) to OD600 = 0.5. Serial peptide dilutions (50 µL) were mixed with 150 µL of bacterial suspension containing 4 µM PI (final in-plate concentration: 3 µM). A high concentration (16µM) of polymyxin B sulfate (polymyxin B; Thermo Fisher) for *E. coli* and 0.1% (v/v) Triton X-100 (Sigma-Aldrich) for *S. aureus* were used as a positive control. Fluorescence was measured every 5 min for 1 h using a microplate reader (VANTAstar) at λ_excitation_ = 530 –15 nm and λ_emission_ = 620 –20 nm^16^.

### LPS-binding using bodipy TR Cadaverine displacement assay

The binding affinity of Hill_BB_C7176 to the lipid A moiety of lipopolysaccharide (produced from *P. aeruginosa* (LPS; Sigma-Aldrich)) was evaluated using a Bodipy TR Cadaverine (BC; Invitrogen) displacement assay. BC, cadaverine linked to the fluorescent dye Bodipy TR, binds to lipid A via electrostatic interactions, leading to a decrease in its fluorescent signal. When BC is displaced from its interaction with lipid A due to competition with other LPS-binding compounds, dequenching of its fluorescence occurs. Serial peptide dilutions (1–32 µM) were prepared in a 50 mM Tris buffer (pH 7.4) in black 96-well plates. LPS (100 ng/mL) and BC (2.5 µM) were added, and fluorescence was recorded every minute over the course of 1 h using a microplate reader (VANTAstar) at λ_excitation_ = 580 –15 nm and λ_emission_ = 620 –20 nm^32,33^.

### *Galleria mellonella* infection model

*Galleria mellonella* larvae (400–450 mg, without signs of melanization) were obtained from Terramania (Arnhem, Netherlands) and kept at room temperature in the dark before experimentation. Two bacterial strains, *S. aureus* and *E. coli*, were used for infection. Each strain was cultured to its mid-logarithmic phase, corresponding to an optical density OD₆₀₀ ≈ 0.4–0.6 for *S. aureus* and *0.5–0.8* for *E. coli*. Bacterial suspensions were prepared by centrifugation at 4,000 × g for 10 min, followed by two washes with sterile 1X PBS. The final inoculum was adjusted to approximately 1 × 10⁶ CFU/mL for *S. aureus* and 1 × 10⁸ CFU/mL for *E. coli* in 1X PBS^34–36^.

For infection, larvae were injected with 20 µL of bacterial suspension into the left hind proleg using a 50 µL Hamilton syringe (Sigma-Aldrich). Thirty minutes post-infection, larvae received treatment in the contralateral hind proleg with either Hill_BB_C7176 (20 µg per larva), the respective reference antibiotic ciprofloxacin HCl (CPX-HCl; Sigma-Aldrich) for *S. aureus* and polymyxin B for *E. coli*, or 1X PBS as a control. Negative control groups included non–infected larvae injected with 1X PBS, non-infected larvae injected with Hill_BB_C7176, and unmanipulated larvae. Following infection and treatment, larvae were incubated at 37 °C in darkness, and survival was monitored at 24, 48, and 72 h to capture the dynamics of the infection process. These time points represent the acute (24 h), subacute (48 h), and late (72 h) phases of infection, allowing for time-dependent assessment of treatment efficacy. Larvae were considered dead if they displayed no response to external stimuli and exhibited full-body melanization. Each experimental group consisted of 30 larvae, pooled from three independent biological replicates (*n* = 3, 10 larvae per replicate).

### Statistical analysis

Statistical analyses were performed using GraphPad Prism 8.0 (GraphPad Software, San Diego, CA, USA). For antimicrobial activity assessments, MIC and MBC values were determined according to Clinical and Laboratory Standards Institute (CLSI) guidelines. IC₅₀ values for cytotoxicity and hemolysis assays were calculated using nonlinear regression analysis. For time-kill kinetics, bacterial counts were averaged across three independent experiments and presented as log10 CFU/mL versus time. Membrane permeabilization data were normalized to negative controls and expressed as relative fluorescence units (RFU). LPS-binding assay results were analyzed as percentage increases in fluorescence relative to baseline measurements. For the *G. mellonella* infection model, Kaplan–Meier survival curves were constructed and analyzed using three statistical methods: Log-rank (Mantel–Cox) test to assess overall survival differences between groups, Log-rank test for trend to evaluate dose-dependent effects, and Gehan–Breslow–Wilcoxon test to give more weight to early time points. A p-value < 0.001 was considered statistically significant for all analyses. All data are presented as mean ± standard deviation (SD) from three significantly independent experiments unless otherwise specified. Survival data are expressed as percentages, with comparisons made between all treatment groups, including PBS and antibiotic controls.

## Results

### Cytotoxicity screening of MRC5-SV2 cells

The cytotoxic effects of the tested peptides were evaluated using the human lung fibroblast cell line MRC5-SV2. The assessment revealed that none of the tested peptides exhibited significant cytotoxic effects at the highest concentration tested (32 µM) (Table 1, Supplementary Table S2).

### Hemolysis activity

To assess the potential hemolytic activity of the peptides, toxicity was evaluated using human red blood cells (Table 1, Supplementary Table S2). The majority of the tested peptides did not induce hemolysis within the tested concentration range. However, for Hill_LB_C16634, the IC₅₀ value obtained in the first repeat was 30.1 µM, while the second repeat yielded an IC₅₀ value > 32 µM. Similarly, for Hill_BB_C7176, the IC₅₀ value in the first screening repeat was 31.6 µM, whereas the second repeat was > 32 µM. All other peptides exhibited IC₅₀ values > 32 µM in both repeats.

### Antimicrobial screening activity results

To evaluate the antimicrobial efficacy of the AMPs, a broad screening was performed against two Gram-positive, two Gram-negative, and two fungal species (Supplementary Table S2). Among the tested peptides, the defensin family demonstrated the most potent antimicrobial activity. Notably, Hill_BB_C7176 exhibited broad-spectrum antibacterial activity against all four bacterial species, demonstrating efficacy against both Gram-negative and Gram-positive bacteria.The MIC values of Hill_BB_C7176 varied across pathogens, with MIC ranges of 2–8 µM against *E. coli*, 8–16 µM against *P. aeruginosa*, and 2–8 µM against *S. aureus*. Other peptides, including Hill_BB_C46948, Hill_AD_C53857, Hill_BB_C3195, and Hill_SB_C1875, showed selective activity against the Gram-negative bacteria *E. coli* and *P. aeruginosa*. Hill_BB_C7985 displayed antimicrobial activity exclusively against *E. coli*. MBC (Supplementary Table S2) values exhibited variability between independent experiments, while the MIC values remained consistent, differing by no more than a factor of two (Table 1).

**Table 1:** Antibacterial Peptides Identified via Dual Screening: IC₅₀, MIC, and Cytotoxicity Profiles; Peptides shown were identified through two independent antibacterial screenings, each initiated at 32 µM. The table reports: IC₅₀ values (µM): Concentration causing 50% inhibition of bacterial growth; MIC values (µM). Cytotoxicity was evaluated on human red blood cells (RBCs) for hemolytic activity and on MRC5-SV2 fibroblasts for general cellular toxicity.

Target type	Target	IC₅₀ and MIC values in µM	Hill_BB_C46948	Hill_BB_C7176	Hill_BB_C1827	Hill_BB_C7985	Hill_C3195	Hill_SB_C1875
Mammalian cells	Human RBCs	IC₅₀	> 32	31.6–>32	> 32	> 32	> 32	> 32
	MRC5-SV2 Cells	IC₅₀	> 32	> 32	> 32	> 32	> 32	> 32
Bacteria	*E. coli*	IC₅₀	1.00–3.57	1.00–4.00	0.82–4.00	3.10–3.97	0.68–1.00	1.00–4.00
		MIC	2–8	2–8	2–8	8	2	2–8
	*P. aeruginosa*	IC₅₀	0.94–1.46	11.37–16.00	> 32	> 32	0.33–0.50	2.11–2.16
		MIC	2	8–16	> 32	> 32	2	8
	*S. aureus*	IC₅₀	> 32	1.37–2.86	> 32	> 32	> 32	> 32
		MIC	> 32	2–8	> 32	> 32	> 32	> 32
	*B. cereus*	IC₅₀	> 32	4.00–4.03	> 32	> 32	> 32	> 32
		MIC	> 32	8	> 32	> 32	> 32	> 32

Fungal susceptibility testing revealed limited activity, with all peptides demonstrating negligible effects against *A. fumigatus* and *C. albicans*, except for Hill_BB_C7985, which exhibited weak activity against *A. fumigatus*.

### Time-kill analysis

**Fig. 1:**
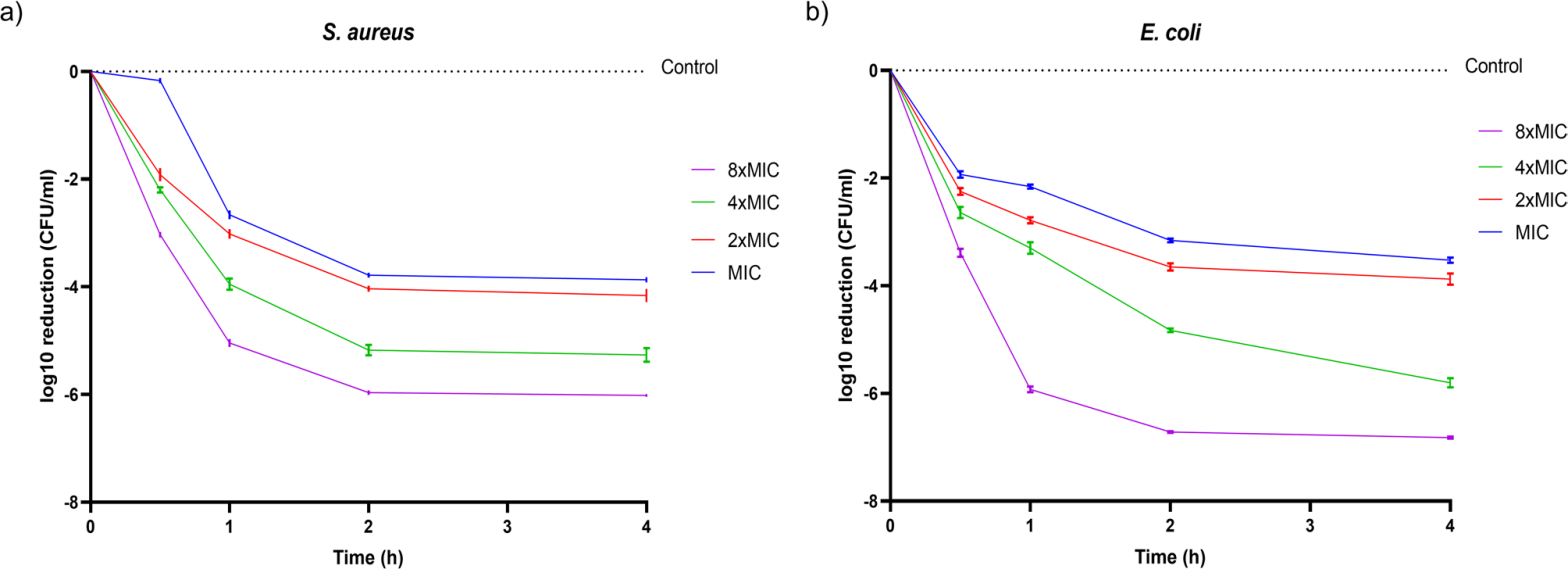
Time-to-kill curves showing log10 reductions in bacterial count after addition of Hill_BB_C7176. (**a**) Killing kinetics of Hill_BB_C7176 at MIC, 2×MIC 4×MIC and 8×MIC against *S. aureus*. (**b**) Killing kinetics of Hill_BB_C7176 at MIC, 2×MIC 4×MIC and 8×MIC against *E. coli*.

For the most potent peptide Hill_BB_C7176, a time-kill experiment was conducted. Results show that Hill_BB_C7176 exhibits potent and rapid bactericidal activity in a concentration-dependent manner against both *S. aureus* (Fig. 1a) and *E. coli* (Fig. 1b). Bactericidal activity, defined as an approximately ≥ 3-log₁₀ reduction in bacterial counts, was achieved within 2 h for *E. coli* at concentrations ≥ 2×MIC (4 µM), and within 1 h for *S. aureus* at ≥ 2×MIC (4µM) or higher. Notably, at 8×MIC (16 µM), complete bacterial eradication (≥ 6-log₁₀ reduction) was observed within 2 h for both *E. coli* and *S. aureus*, with no evidence of regrowth over the duration of the experiment. These results demonstrate the peptide rapid and effective killing kinetics against Gram-positive and Gram-negative bacteria.

### Inner membrane permeabilization by the propidium iodide uptake assay results

**Fig. 2:**
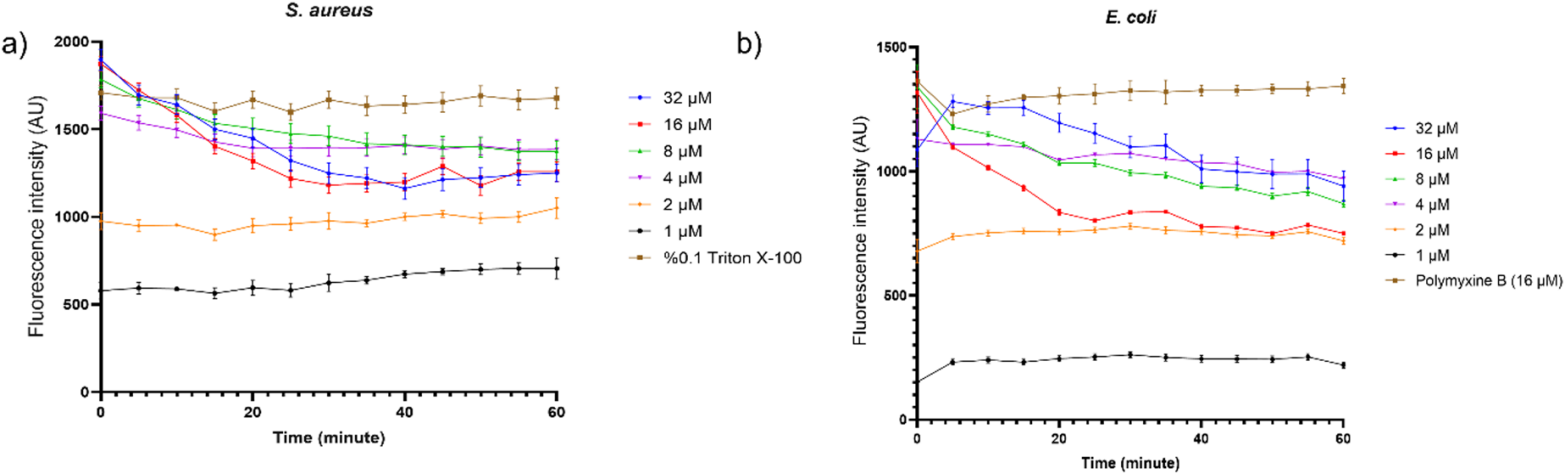
Fluorescence caused by propidium iodide uptake in *S. aureus* (**a**) and *E. coli* (**b**) after addition of Hill_BB_C7176. Values were normalized with the negative control. AU: arbitrary units. The assay was carried out in biological triplicate. The mean ± standard deviation is given.

The ability of the peptides to permeabilize bacterial membranes was assessed using a PI uptake assay in *S. aureus* (Fig. 2a) and *E. coli* (Fig. 2b). Fluorescence intensity was normalized to a negative control. PI uptake occurred rapidly upon exposure to Hill_BB_C7176, with a sharp increase in fluorescence followed by signal stabilization^37^. PI is a DNA-intercalating dye that cannot traverse intact bacterial membranes; thus, its uptake is indicative of membrane permeabilization. The permeabilization effect was concentration-dependent, with low signals observed below 2 µM. Notably, Hill_BB_C7176 caused a greater increase in fluorescence for *S. aureus* compared to *E. coli*, indicating higher PI uptake across the cell membrane for Gram-positive bacteria.

### LPS-binding using bodipy TR cadaverine displacement assay

**Fig. 3:**
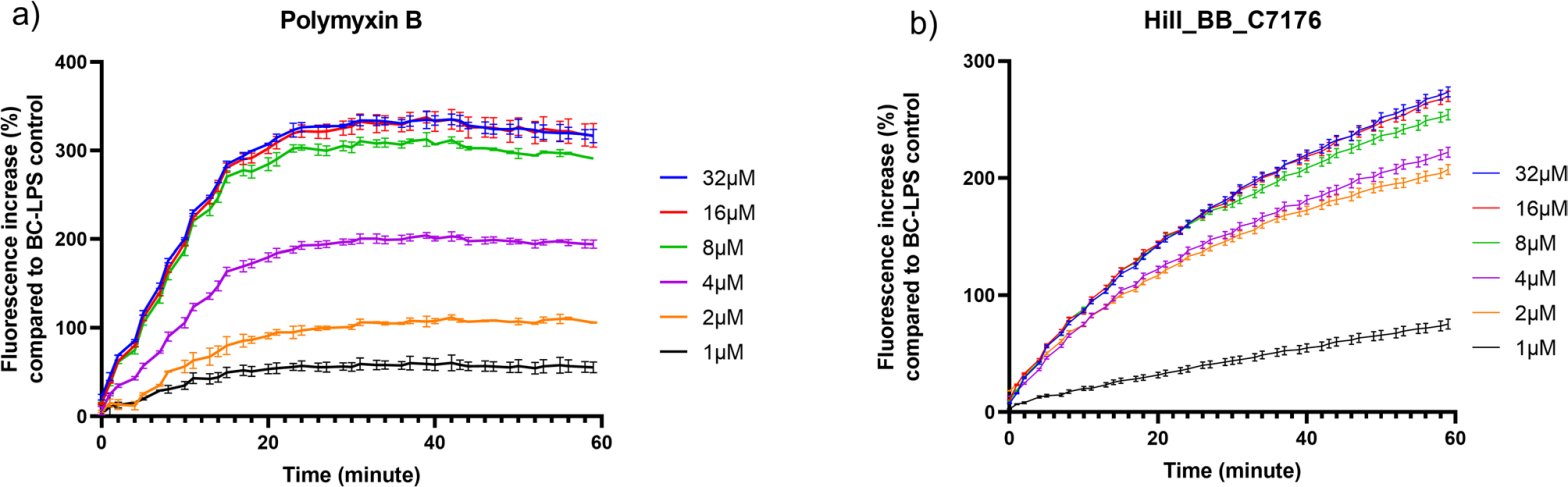
Bodipy cadaverine (BC) displacement assay for polymyxin B and Hill_BB_C7176. (**a**) Fluorescence caused by polymyxin B-LPS interactions. (**b**) Fluorescence caused by Hill_BB_C7176-LPS interactions. As a control, samples with BC and LPS (100 ng/mL) without peptide treatment were included. The assay was carried out in biological triplicate. The mean ± standard deviation is given.

To study the affinity of the AMP to the Lipid A toxic center of LPS, a fluorescent displacement assay using BC was carried out. Hill_BB_C7176 shows a concentration-dependent increase in fluorescence, with an approximately 300% increase at 32 µM, indicating strong binding to lipid A. A comparable increase in fluorescence is observed for polymyxin B, a peptide antibiotic that binds the phosphate groups of LPS and uses it as an entry point to cross the membranes of Gram-negative bacteria (Fig. 3)^32,38^. Hill_BB_C7176 showed a gradual BC displacement, requiring a longer period to reach peak fluorescence levels compared to polymyxin B which achieved a plateau phase within the first 20 min of lipid A contact (Fig. 3).

### Survival analysis of ***Galleria mellonella*** larvae infected with ***S. aureus*** and ***E. coli***

**Fig. 4:**
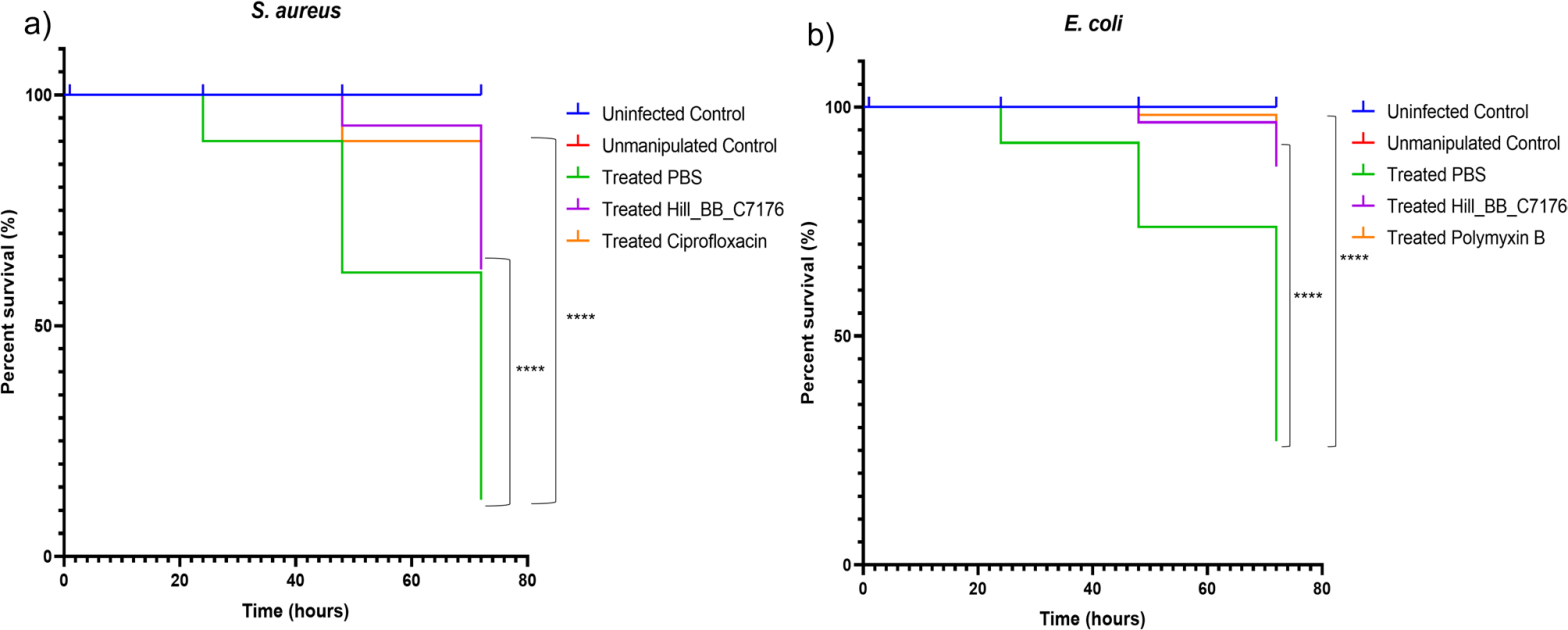
Kaplan-Meier survival curves of *Galleria mellonella* larvae infected with (**a**) *S. aureus*, and (**b**) *E. coli*, treated post-infection with Hill_BB_C7176 or antibiotics. Larvae were monitored over 72 h (*n* = 30 per group). Significant differences between treatment groups were evaluated using the log-rank test (****=*p* < 0.0001).

To evaluate the *in vivo* antimicrobial efficacy of Hill_BB_C7176, a *Galleria mellonella* infection model using *S. aureus* and *E. coli* was carried out. Larval survival was monitored every 24 h over a 72-hour period post-infection and treatment. As shown in Fig. 4a, S. *aureus*-infected larvae treated with Hill_BB_C7176 exhibited markedly enhanced survival compared to the PBS-treated infected group. While the survival rate in the Hill_BB_C7176 group reached approximately 60%, ciprofloxacin treatment resulted in around 90% survival. Nevertheless, Hill_BB_C7176 still provided significant *in vivo* protection. In contrast, the PBS treated infected larvae showed a significant decline in survival, with only a minority of larvae surviving past 48 h. The uninfected control and unmanipulated groups maintained near 100% survival throughout the experiment.

Similarly, in the *E. coli* infection model (Fig. 4b), treatment with Hill_BB_C7176 significantly improved larval survival compared to the infected larvae. Notably, the survival curves for the Hill_BB_C7176 and polymyxin B groups showed similar trends, indicating that the peptide activity in this model aligns with that of the reference peptide antibiotic at the tested concentrations. Infected larvae demonstrated a sharp decline in survival over 72 h, highlighting the protective effect of Hill_BB_C7176. Survival analyses showed statistically significant differences among treatment groups for both *S. aureus* and *E. coli* (*p* < 0.0001).

## Discussion

Among the tested peptides, Hill_BB_C7176 and Hill_BB_C3195 displayed the most promising antimicrobial profiles, with low MIC values against a range of bacterial pathogens, including *S. aureus*, *E. coli*, *P. aeruginosa*, and *B. cereus*. These peptides showed consistent inhibitory and bactericidal activity across experimental repeats, with MIC values as low as 0.33–2 µM, indicating strong potency, particularly against Gram-negative bacteria. Several other peptides, such as Hill_BB_C7985, Hill_SB_C1875, and Hill_BB_C1827 showed moderate antimicrobial effects, mainly against *E. coli* and *P. aeruginosa*, but with higher variability or less consistent results. In contrast, peptides including NHill_AD_C53857, NHill_AD_C49215, Hill_BB_C6571, and Hill_LB_C16634 did not show any significant antimicrobial activity under the tested conditions, with MIC and MBC values above 32 µM. The differential antimicrobial activity observed across the library can be attributed to physicochemical variation among the peptides. Hill_BB_C7176 possesses a high net positive charge (+ 5 to + 8) and a well-defined amphipathic profile, which are hallmarks of biologically active defensins^18,39^. In contrast, the inactive peptides in our library (e.g., NHill_AD_C53857) lacked sufficient cationic charge, possibly preventing the electrostatic interaction required to overcome the bacterial cell wall barrier.

Importantly, all peptides demonstrated negligible hemolytic activity against human red blood cells and MRC5-SV2 lung fibroblast cells, For most peptides, IC₅₀ values exceeded 32 µM in both experimental repeats. However, Hill_LB_C16634 showed an IC₅₀ of 30.1 µM in the first repeat and > 32 µM in the second repeat, while Hill_BB_C7176 exhibited 31.6 µM in the first repeat and > 32 µM in the second. All other peptides consistently displayed IC₅₀ values > 32 µM across both repeats. These findings show that the active peptides, particularly Hill_BB_C7176 and Hill_BB_C3195, possess both antimicrobial efficacy and a favorable *in vitro* safety profile, making them interesting AMP candidates for further research. Interestingly, Hill_BB_C7176 exhibited a broader antibacterial spectrum than Hill-Def4, a sequence analog previously reported by Van Moll *et al.* with activity only against Gram-negative bacteria (Supplementary Table S3). Antimicrobial peptide activity is known to be influenced by multiple experimental factors^16^. Variations in assay conditions, including media composition, inoculum size, and incubation time, can affect peptide–pathogen interactions and may partially account for differences in MIC or MBC values across studies^40,41^. In addition, differences in peptide synthesis and handling may introduce further variability. Even when synthesizing identical sequences, peptides may differ in physicochemical properties due to batch-specific factors such as incomplete deprotection, residual by-products, or variations in purification efficiency and folding^21,42^. These findings underscore the importance of standardization of experimental set-up within AMP research.

Due to its broad-spectrum, potent antibacterial activity, Hill_BB_C7176, was chosen for further experimental investigation. Time-kill experiments confirmed its rapid, concentration-dependent antibacterial activity. Bactericidal activity (≥ 3-log₁₀ reduction in bacterial counts) and bacterial eradication (≥ 6-log₁₀ reduction) were achieved within the first two hours of incubation at supra-MIC concentrations for both *E. coli* and *S. aureus.*

To obtain exploratory insight into the interaction of Hill_BB_C7176 with the bacterial membrane, PI uptake and BC displacement assays were employed. The ability to quickly permeabilize bacterial membranes suggests a direct interaction with lipid bilayers, leading to cell death, as confirmed by the PI uptake assay. Given that PI uptake occurred rapidly (within 5 min) and was detectable at sublethal concentrations. However, these findings do not define a definitive or exclusive mechanism of action; rather, they provide evidence of membrane-peptide interactions^43^. The higher PI uptake in *S. aureus* compared to *E. coli* likely reflects the absence of an outer membrane in Gram-positive bacteria. Despite the presence of a thicker peptidoglycan layer, peptides may gain access to the cytoplasmic membrane more readily in *S. aureus* than in *E. coli.* While the observed differences in antibacterial activity are likely influenced by variations in cell membrane structure, the present study does not directly establish the specific mechanistic basis underlying these species-specific differences, necessitating further in-depth characterization.

The binding affinity of Hill_BB_C7176 to lipid A suggests that LPS functions as a potential entry point in the outer cell membrane of Gram-negative bacteria. Lipid A is a conserved component of LPS in the outer membrane of Gram-negative bacteria, and peptides that effectively interact with it can destabilize the membrane and enhance bactericidal action. The specificity of the interaction between Hill_BB_C7176 and Lipid A is evidenced by the concentration-dependent nature of the fluorescence increase. In the BC displacement assay, fluorescence dequenching only occurs when a competitor possesses a higher affinity for the lipid A binding site than the BC probe itself. Hill_BB_C7176 achieved a 300% increase in fluorescence, a profile similar to the well-characterized LPS-binding antibiotic polymyxin B. The potent activity of Hill_BB_C7176 against Gram-positive organisms such as *S. aureus*, however, suggests an alternative initial interaction pathway. Cationic AMPs typically interact with negatively charged components of the Gram-positive cell wall, such as lipoteichoic acid^4,9^. These electrostatic interactions could facilitate the accumulation of Hill_BB_C7176 on the thick, porous peptidoglycan layer, allowing it to subsequently reach and disrupt the cytoplasmic membrane, as evidenced by our PI uptake results.

Interestingly, while Hill_BB_C7176 exhibited potent antibacterial activity against *E. coli*, its efficacy was markedly reduced against *P. aeruginosa*. The differential activity between the two Gram-negative bacteria likely reflects differences in their outer membrane architecture^44,45^. *P. aeruginosa* has a less permeable outer leaflet with increased LPS crosslinking compared to *E. coli*, which can limit the accessibility of cationic AMPs. Extensive crosslinking within the LPS layer contributes to a strong permeability barrier, which could reduce the binding and penetration of Hill_BB_C7176^46^. Overall, these mechanistic findings should be interpreted with caution. While our data support strong Lipid A affinity and membrane permeability, they do not conclusively demonstrate the AMP mode of action nor therapeutic equivalence with known antibiotics such as Polymyxin B^32^. Further structural modelling and mode of action studies will be required to fully elucidate the specific structure-function relationships of Hill_BB_C7176.

Finally, *in vivo* efficacy was validated using the *Galleria mellonella* infection model, where Hill_BB_C7176 significantly improved survival in larvae infected with either *S. aureus* or *E. coli*. This finding is consistent with the peptide *in vitro* activity profile and supports its potential against both Gram-positive and Gram-negative bacteria. However, while the *G. mellonella* model provides a valuable preliminary *in vivo *system due to its simplicity and presence of innate immune responses, it is essential to emphasize that as an invertebrate model, it cannot directly predict mammalian pharmacokinetics or clinical efficacy. Thus, although the model allows a first assessment of efficacy and toxicity, further validation in vertebrate systems is necessary to fully evaluate the clinical potential of Hill_BB_C7176. Within our study, time points were carefully tailored to match the biological context and kinetics of each assay. For instance, the PI uptake and LPS-binding assays were designed to capture rapid, early molecular events, and thus applied shorter time frames (e.g., 0–60 min). In contrast, assays such as the cytotoxicity screening and *G. mellonella* infection model required longer observation periods (up to 72 h) to assess cumulative effects on cell viability and organism survival.

Despite the promising antimicrobial and preliminary *in vivo* results, several limitations must be acknowledged. First, while Hill_BB_C7176 showed efficacy in an invertebrate model (*G. mellonella*), mammalian biological data are currently lacking to fully assess its therapeutic safety and systemic behavior. Secondly, further assays, such as serum stability tests, are necessary to determine the peptide durability in a complex physiological environment. Finally, our study focused on a restricted number of Gram-negative and Gram-positive strains; expanding the screening to a broader panel of bacterial isolates will be essential to define the full spectrum of activity of these BSF-derived peptides. In summary, Hill_BB_C7176 appears to exhibit broad-spectrum antibacterial activity, rapid bactericidal effects, membrane-disruptive properties, and *in vivo* efficacy, alongside low cytotoxicity *in vitro*. These preliminary findings support the further research on the antibacterial activity of Hill_BB_C7176.

## Conclusion

This study provides a comprehensive evaluation of the antimicrobial and cytotoxic properties of selected AMPs. Hill_BB_C7176 emerged as a highly effective antimicrobial peptide with broad-spectrum antibacterial activity and a rapid bactericidal effect. Notably, a promising BSF AMP was identified due to its dual action against Gram-positive bacteria such as *S. aureus* and Gram-negative pathogens, including *E. coli* and *P. aeruginosa*. Its ability to permeabilize bacterial membranes further supports its potential as an antimicrobial lead molecule. While its moderate hemolytic activity warrants further investigation, Hill_BB_C7176 remains a promising candidate for future antibacterial development. Future studies should focus on assessing mammalian *in vivo* efficacy and toxicity, peptide stability in physiological conditions, and structural optimization strategies to improve the AMP activity profile.

## Supplementary Information

Below is the link to the electronic supplementary material.

Supplementary Material 1

Supplementary Material 2

Supplementary Material 3

## Data Availability

The datasets used and/or analysed during the current study are available from the corresponding author on reasonable request.

## References

[1] Murray, C. J. et al. Global burden of bacterial antimicrobial resistance in 2019: a systematic analysis. *Lancet* **399**, 629–655. 10.1016/S0140-6736(21)02724-0 (2022). 35065702 10.1016/S0140-6736(21)02724-0 PMC8841637

[2] World Health Organization. Global antimicrobial resistance and use surveillance system (GLASS) report. Geneva: (2023).

[3] Cabrera-Aguas, M., Chidi-Egboka, N., Kandel, H. & Watson, S. L. Antimicrobial resistance in ocular infection: A review. *Clin. Exp. Ophthalmol.* **52**, 258–275. 10.1111/ceo.14377 (2024). 38494451 10.1111/ceo.14377

[4] Moravej, H. et al. Antimicrobial peptides: features, action, and their resistance mechanisms in bacteria. *Microb. Drug Resist.* **24**, 747–767. 10.1089/mdr.2017.0392 (2018). 29957118 10.1089/mdr.2017.0392

[5] Lei, J. et al. The antimicrobial peptides and their potential clinical applications. *Am. J. Transl Res.* **11**, 3919–3931 (2019). 31396309 PMC6684887

[6] , L. C. F.,, F. C. M. &, S. S. D. Intracellular targeting mechanisms by antimicrobial peptides. *Antimicrob. Agents Chemother.* **61**10.1128/AAC.02340-16 (2017). 10.1128/AAC.02340-16 PMC5365711 28167546

[7] Neghabi Hajigha, M. et al. Antiviral and antibacterial peptides: Mechanisms of action. *Heliyon* **10**10.1016/j.heliyon.2024.e40121 (2024). 10.1016/j.heliyon.2024.e40121 PMC11693924 39748995

[8] De Stefano, F. et al. Secondary products and bioactive compounds of *Hermetia illucens*: Extraction, chemical properties, and potential application of antimicrobial peptides. The Black Soldier Fly (Hermetia illucens), 221–56. (Elsevier, 2026). 10.1016/B978-0-443-29896-7.00004-4

[9] Zasloff, M. Antimicrobial peptides of multicellular organisms. *Nature* **415**. (2002). 10.1038/415389a 11807545

[10] Bulet, P., Stöcklin, R. & Menin, L. Anti-microbial peptides: From invertebrates to vertebrates. *Immunol. Rev.* **198**, 169–184. 10.1111/j.0105-2896.2004.0124.x (2004). 15199962 10.1111/j.0105-2896.2004.0124.x

[11] De Mandal, S. et al. Antimicrobial peptides: novel source and biological function with a special focus on entomopathogenic Nematode/Bacterium symbiotic complex. *Front. Microbiol.* 12. 10.3389/fmicb.2021.555022 (2021). 10.3389/fmicb.2021.555022 PMC8318700 34335484

[12] Manniello, M. D. et al. Insect antimicrobial peptides: potential weapons to counteract the antibiotic resistance. *Cell. Mol. Life Sci.* **78**, 4259–4282. 10.1007/s00018-021-03784-z (2021). 33595669 10.1007/s00018-021-03784-z PMC8164593

[13] Elhag, O. et al. Screening, expression, purification and functional characterization of novel antimicrobial peptide genes from hermetia illucens (L). *PLoS One*. 12. 10.1371/journal.pone.0169582 (2017). 10.1371/journal.pone.0169582 PMC5215879 28056070

[14] Park, S. & Yoe, S. M. A novel cecropin-like peptide from black soldier fly, *Hermetia illucens*: Isolation, structural and functional characterization: A cecropin-like peptide from H. illucens. *Entomol. Res.* **47**, 115–124. 10.1111/1748-5967.12226 (2017).

[15] Vogel, H., Müller, A., Heckel, D. G., Gutzeit, H. & Vilcinskas, A. Nutritional immunology: Diversification and diet-dependent expression of antimicrobial peptides in the black soldier fly Hermetia illucens. *Dev. Comp. Immunol.* **78**, 141–148. 10.1016/j.dci.2017.09.008 (2018). 28966127 10.1016/j.dci.2017.09.008

[16] Van Moll, L. et al. *In vitro* evaluation of antimicrobial peptides from the Black Soldier Fly (Hermetia Illucens) against a selection of human pathogens. *Microbiol. Spectr.* **10**, 1–17 (2022). 10.1128/spectrum.01664-21 PMC8729770 34985302

[17] Moretta, A. et al. Antimicrobial peptides: A new hope in biomedical and pharmaceutical fields. *Front. Cell. Infect. Microbiol.* 11. 10.3389/fcimb.2021.668632 (2021). 10.3389/fcimb.2021.668632 PMC8238046 34195099

[18] Moretta, A. et al. A bioinformatic study of antimicrobial peptides identified in the Black Soldier Fly (BSF) *Hermetia illucens* (Diptera: Stratiomyidae). *Sci. Rep.* 10. 10.1038/s41598-020-74017-9 (2020). 10.1038/s41598-020-74017-9 PMC7547115 33037295

[19] Waghu, F. H., Barai, R. S., Gurung, P. & Idicula-Thomas, S. CAMPR3: a database on sequences, structures and signatures of antimicrobial peptides. *Nucleic Acids Res.* **44**, D1094–D1097. 10.1093/nar/gkv1051 (2016). 26467475 10.1093/nar/gkv1051 PMC4702787

[20] Gawde, U. et al. CAMPR4: a database of natural and synthetic antimicrobial peptides. *Nucleic Acids Res.* **51**, D377–D383. 10.1093/nar/gkac933 (2023). 36370097 10.1093/nar/gkac933 PMC9825550

[21] Behrendt, R., White, P. & Offer, J. Advances in Fmoc solid-phase peptide synthesis. *J. Pept. Sci.* **22**, 4–27. 10.1002/psc.2836 (2016). 26785684 10.1002/psc.2836 PMC4745034

[22] Cos, P., Vlietinck, A. J., Berghe, D. & Vanden, Maes, L. Anti-infective potential of natural products: How to develop a stronger *in vitro* ‘proof-of-concept’. *J. Ethnopharmacol.* **106**, 290–302. 10.1016/j.jep.2006.04.003 (2006). 16698208 10.1016/j.jep.2006.04.003

[23] Kuete, V., Karaosmanoğlu, O. & Sivas, H. Anticancer Activities of African Medicinal Spices and Vegetables*. Medicinal Spices and Vegetables from Africa* 271–297 (Elsevier, 2017). 10.1016/B978-0-12-809286-6.00010-8

[24] Wang, S., Liu, X. Q., Kang, O. H. & Kwon, D. Y. Combination of sanguisorbigenin and conventional antibiotic therapy for methicillin-resistant staphylococcus aureus: inhibition of biofilm formation and alteration of cell membrane permeability. *Int. J. Mol. Sci.* **23**10.3390/ijms23084232 (2022). 10.3390/ijms23084232 PMC9032919 35457049

[25] Vishnepolsky, B. et al. *De novo* design and *in vitro* testing of antimicrobial peptides against gram-negative bacteria. *Pharmaceuticals* **12**10.3390/ph12020082 (2019). 10.3390/ph12020082 PMC6631481 31163671

[26] Monteiro, C. et al. A 17-mer membrane-active MSI-78 derivative with improved selectivity toward bacterial cells. *Mol. Pharm.* **12**, 2904–2911. 10.1021/acs.molpharmaceut.5b00113 (2015). 26066462 10.1021/acs.molpharmaceut.5b00113

[27] Win, T. S. et al. HemoPred: A web server for predicting the hemolytic activity of peptides. *Future Med. Chem.* **9**, 275–291. 10.4155/fmc-2016-0188 (2017). 28211294 10.4155/fmc-2016-0188

[28] Sæbø, I. P., Bjørås, M., Franzyk, H., Helgesen, E. & Booth, J. A. Optimization of the hemolysis assay for the assessment of cytotoxicity. *Int. J. Mol. Sci.* **24**10.3390/ijms24032914 (2023). 10.3390/ijms24032914 PMC9917735 36769243

[29] Zhang, S., Zhang, R., Li, C., Cong, H. & Yu, B. Screening of an antibacterial and coagulation multifunctional peptide and Its application in wound healing. *Chem. – Eur. J.* **31**10.1002/chem.202403214 (2025). 10.1002/chem.202403214 39673092

[30] Mascio, C. T. M., Alder, J. D. & Silverman, J. A. Bactericidal action of daptomycin against stationary-phase and nondividing *Staphylococcus aureus* cells. *Antimicrob. Agents Chemother.* **51**, 4255–4260. 10.1128/AAC.00824-07 (2007). 17923487 10.1128/AAC.00824-07 PMC2167999

[31] DassanayakeRP, FalkenbergSM, BriggsRE, TatumFM & SaccoRE Antimicrobial activity of bovine NK-lysin-derived peptides on bovine respiratory pathogen Histophilus somni. *PLoS One*. **12**10.1371/journal.pone.0183610 (2017). 10.1371/journal.pone.0183610 PMC5565109 28827826

[32] Van Moll, L. et al. In-depth biological characterization of two black soldier fly anti-*Pseudomonas* peptides reveals LPS-binding and immunomodulating effects. *MSphere* **8**10.1128/msphere.00454-23 (2023). 10.1128/msphere.00454-23 PMC10597467 37800918

[33] Kim, E. Y., Kumar, S. D., Bang, J. K. & Shin, S. Y. Mechanisms of antimicrobial and antiendotoxin activities of a triazine-based amphipathic polymer. *Biotechnol. Bioeng.* **117**, 3508–3521. 10.1002/bit.27499 (2020). 32662872 10.1002/bit.27499

[34] Alnezary, F. S., Almutairi, M. S., Alhifany, A. A. & Almangour, T. A. Assessing *Galleria mellonella* as a preliminary model for systemic *Staphylococcus aureus *infection: Evaluating the efficacy and impact of vancomycin and *Nigella sativa *oil on gut microbiota. *Saudi Pharm. J.* **31**, 101824. 10.1016/j.jsps.2023.101824 (2023). 37965487 10.1016/j.jsps.2023.101824 PMC10641552

[35] Ménard, G. et al. *Galleria mellonella* larvae as an infection model to investigate sRNA-mediated pathogenesis in *Staphylococcus aureus*. *Front. Cell. Infect. Microbiol.* 11. 10.3389/fcimb.2021.631710 (2021). 10.3389/fcimb.2021.631710 PMC8089379 33954118

[36] Alghoribi, M. F., Gibreel, T. M., Dodgson, A. R., Beatson, S. A. & Upton, M. *Galleria mellonella *infection model demonstrates high lethality of ST69 and ST127 uropathogenic * E. coli.**PLoS One*. **9**, e101547. 10.1371/journal.pone.0101547 (2014). 25061819 10.1371/journal.pone.0101547 PMC4111486

[37] Sautrey, G. et al. Negatively charged lipids as a potential target for new amphiphilic aminoglycoside antibiotics: A biophysical study. *J. Biol. Chem.* **291**, 13864–13874. 10.1074/jbc.M115.665364 (2016). 27189936 10.1074/jbc.M115.665364 PMC4919468

[38] Goode, A., Yeh, V. & Bonev, B. B. Interactions of polymyxin B with lipopolysaccharide-containing membranes. *Faraday Discuss.* **232**, 317–329. 10.1039/D1FD00036E (2021). 34550139 10.1039/d1fd00036e PMC8704168

[39] Du, K. et al. Optimized charge/hydrophobicity balance of antimicrobial peptides against polymicrobial abdominal infections.* Macromol. Biosci.* 24. 10.1002/mabi.202300451 (2024) 10.1002/mabi.202300451 37997560

[40] Hancock, R. E. W. & Sahl, H. G. Antimicrobial and host-defense peptides as new anti-infective therapeutic strategies. *Nat. Biotechnol.* **24**, 1551–1557. 10.1038/nbt1267 (2006). 17160061 10.1038/nbt1267

[41] Wang, G., Li, X. & Wang, Z. APD3: The antimicrobial peptide database as a tool for research and education. *Nucleic Acids Res.* **44**, D1087–D1093. 10.1093/nar/gkv1278 (2016). 26602694 10.1093/nar/gkv1278 PMC4702905

[42] Merrifield, R. B. & City, Y. Solid-phase peptide synthesis. (1969). 10.1002/9780470122778.ch6 4307033

[43] Scocchi, M., Mardirossian, M., Runti, G. & Benincasa, M. Non-membrane permeabilizing modes of action of antimicrobial peptides on bacteria. *Curr. Top. Med. Chem.* **16**, 76–88. 10.2174/1568026615666150703121009 (2016). 26139115 10.2174/1568026615666150703121009

[44] Frirdich, E., Whitfield, C. & Review Lipopolysaccharide inner core oligosaccharide structure and outer membrane stability in human pathogens belonging to the Enterobacteriaceae. *J. Endotoxin Res.* **11**, 133–144. 10.1177/09680519050110030201 (2005). 15949142 10.1179/096805105X46592

[45] Ruan, X., Monjarás Feria, J., Hamad, M. & Valvano, M. A. *Escherichia coli* and *Pseudomonas aeruginosa* lipopolysaccharide O-antigen ligases share similar membrane topology and biochemical properties. *Mol. Microbiol.* **110**, 95–113. 10.1111/mmi.14085 (2018). 30047569 10.1111/mmi.14085

[46] Kreamer, N. N. K. et al. Acylated-acyl carrier protein stabilizes the *Pseudomonas aeruginosa* WaaP lipopolysaccharide heptose kinase. *Sci. Rep.* **8**, 14124. 10.1038/s41598-018-32379-1 (2018). 30237436 10.1038/s41598-018-32379-1 PMC6147952

