# Supplementary material for: Antimicrobial effects of novel Hermetia illucens peptides
Source: Sci Rep. 2026 Feb 24;16:10398. doi: 10.1038/s41598-026-40997-3 (PMC13031288; doi:10.1038/s41598-026-40997-3)
Supplement: Supplementary file 1 — Supplementary Material 1 [file 41598_2026_40997_MOESM1_ESM.docx]

**Supplementary Table 1** Overview of the selected Black Soldier Fly peptides, their structural class, primary sequence, molecular weight, and purity. For the antimicrobial screening, peptides were dissolved in either demineralized water or DMSO, depending on their water solubility.

| Peptides | Sequences | Length  (aa) | Molecular weight (g/mol) | Total hydrophobic ratio | Isoelectric point | Solvent | Purity (%w/w) | The total net charge |
| --- | --- | --- | --- | --- | --- | --- | --- | --- |
| Hill_BB_C46948 (Knottin) | RKCTASQCTRVCKKLGYKRGYCQSSTKCVC | 30 | 3390.071 | 33 | 9.64 | ddH_2_O | > 95 | +8 |
| Hill_LB_C16634 (Knottin) | IKCTASICTQICRILKYKCGYCASASRCVCLK | 32 | 3531.395 | 53 | 9.18 | DMSO | > 95 | +6 |
| NHill_AD_C53857 (Alo) | CINNGDGCQPDGRQGNCCSGYCHKEPGWVTGYCR | 34 | 3679.079 | 26 | 6.70 | DMSO | > 95 | + 0.25 |
| NHill_AD_C49215 (Alo) | CIANGNGCQPDGRQGNCCSGFCYKQRGWVAGYCRRR | 36 | 3985.541 | 33 | 9.18 | DMSO | > 95 | +5 |
| Hill_BB_C6571 (Defensin) | ATCTNWNCRTQCIARGKRGGYCVERNICKCTS | 32 | 3597.190 | 37 | 9.18 | DMSO | > 95 | +5 |
| Hill_BB_C7176 (Defensin) | ATCDLLSPFKVGHAACALHCIALGRRGGWCDGRAVCNCRR | 40 | 4259.049 | 52 | 8.98 | DMSO | > 95 | +4.5 |
| Hill_BB_C1827 (Defensin) | TTCTHLNCKLHCVLYRKRSGRCDRFNICKCI | 31 | 3686.457 | 41 | 9.38 | DMSO | > 95 | + 6.5 |
| Hill_BB_C7985 (Defensin) | FTCSNLGCKAQCIILGNRSGGCNRLGVCQCN | 31 | 3233.819 | 45 | 8.70 | DMSO | > 95 | +3 |
| Hill_C3195 (Cecropin) | GWWKKVFKPVEKLGQRVRDAGIQGIAIAQQGANVLATVRGGPPQ | 44 | 4726.506 | 40 | 11.07 | ddH_2_O | > 95 | +6 |
| Hill_ SB_C1875 (Cecropin) | GQGESRSLWKKIFKPVEKLGQRVRDAGIQGIAIAQQGANVLATVRGGPPQ | 50 | 5312.123 | 36 | 11.00 | ddH_2_O | > 95 | +5 |
