# Supplementary material for: Antimicrobial effects of novel Hermetia illucens peptides
Source: Sci Rep. 2026 Feb 24;16:10398. doi: 10.1038/s41598-026-40997-3 (PMC13031288; doi:10.1038/s41598-026-40997-3)
Supplement: Supplementary file 2 — Supplementary Material 2 [file 41598_2026_40997_MOESM2_ESM.docx]

**Supplementary Table 2** IC50 values (concentrations leading to 50% growth inhibition of the pathogen), MIC values, and MBC values; obtained in two individual screenings starting at 32 µM (fungi obtained from one individual screening). Peptides that exhibited antimicrobial activity, low cytotoxicity, and no hemolytic effect are highlighted in bold.

| AMPs | Repeat | Half maximal inhibitory concentration (IC50, µM) | | | | | | | | Minimum Inhibitory Concentration (MIC, µM) | | | | | | Minimum Bactericidal Concentration (MBC, µM) | | | | | |
| --- | --- | --- | --- | --- | --- | --- | --- | --- | --- | --- | --- | --- | --- | --- | --- | --- | --- | --- | --- | --- | --- |
|  |  | Red blood cells (human) | MRC5-SV2 cells | *C. albicans* | *A. fumigatus* | *E. coli* | *P. aeruginosa* | *S. aureus* | *B. cereus* | *C. albicans* | *A. fumigatus* | *E. coli* | *P. aeruginosa* | *S. aureus* | *B. cereus* | *C. albicans* | A. fumigatus | *E. coli* | *P. aeruginosa* | *S. aureus* | *B. cereus* |
| Hill_BB_C46948 | 1 | >32 | >32 | >32 | >32 | **3,57** | **1,46** | >32 | >32 | >32 | >32 | **8** | **2** | >32 | >32 | >32 | >32 | **8** | **2** | >32 | >32 |
|  | 2 | >32 | >32 | >32 | >32 | **1** | **0,94** | >32 | >32 | >32 | >32 | **2** | **2** | >32 | >32 | >32 | >32 | **8** | **8** | >32 | >32 |
| Hill_LB_C16634 | 1 | **30,1** | >32 | >32 | >32 | >32 | >32 | >32 | >32 | >32 | >32 | >32 | >32 | >32 | >32 | >32 | >32 | >32 | >32 | >32 | >32 |
|  | 2 | >32 | >32 | >32 | >32 | >32 | >32 | >32 | >32 | >32 | >32 | >32 | >32 | >32 | >32 | >32 | >32 | >32 | >32 | >32 | >32 |
| NHill_AD_C53857 | 1 | >32 | >32 | >32 | >32 | >32 | >32 | >32 | >32 | >32 | >32 | >32 | >32 | >32 | >32 | >32 | >32 | >32 | >32 | >32 | >32 |
|  | 2 | >32 | >32 | >32 | >32 | >32 | >32 | >32 | >32 | >32 | >32 | >32 | >32 | >32 | >32 | >32 | >32 | >32 | >32 | >32 | >32 |
| NHill_AD_C49215 | 1 | >32 | >32 | >32 | >32 | >32 | >32 | >32 | >32 | 32 | >32 | >32 | >32 | >32 | >32 | 32 | >32 | >32 | >32 | >32 | >32 |
|  | 2 | >32 | >32 | >32 | >32 | >32 | >32 | >32 | >32 | >32 | >32 | >32 | >32 | >32 | >32 | >32 | >32 | >32 | >32 | >32 | >32 |
| Hill_BB_C6571 | 1 | >32 | >32 | >32 | >32 | >32 | >32 | >32 | >32 | >32 | >32 | >32 | >32 | >32 | >32 | >32 | >32 | >32 | >32 | >32 | >32 |
|  | 2 | >32 | >32 | >32 | >32 | >32 | >32 | >32 | >32 | >32 | >32 | >32 | >32 | >32 | >32 | >32 | >32 | >32 | >32 | >32 | >32 |
| Hill_BB_C7176 | 1 | **31,6** | >32 | >32 | >32 | **4** | **11,37** | **1,37** | **4** | >32 | >32 | **8** | **8** | **2** | **8** | >32 | >32 | **8** | **8** | **8** | **8** |
|  | 2 | >32 | >32 | >32 | >32 | **1,00** | **14,16** | **2,86** | **4,03** | >32 | >32 | **2** | **16** | **8** | **8** | >32 | >32 | **8** | **8** | **8** | **8** |
| Hill_BB_C1827 | 1 | >32 | >32 | >32 | >32 | **4** | >32 | >32 | >32 | >32 | >32 | **8** | >32 | >32 | >32 | >32 | >32 | >32 | >32 | >32 | >32 |
|  | 2 | >32 | >32 | >32 | >32 | **0,82** | >32 | >32 | >32 | >32 | >32 | **2** | >32 | >32 | >32 | >32 | >32 | >32 | >32 | >32 | >32 |
| Hill_BB_C7985 | 1 | >32 | >32 | >32 | **30,34** | **3,97** | >32 | >32 | >32 | >32 | >32 | **8** | >32 | >32 | >32 | >32 | >32 | >32 | >32 | >32 | >32 |
|  | 2 | >32 | >32 | >32 | >32 | **3,10** | >32 | >32 | >32 | >32 | >32 | **8** | >32 | >32 | >32 | >32 | >32 | **8** | >32 | >32 | >32 |
| Hill_BB_C3195 | 1 | >32 | >32 | >32 | >32 | **1** | **0,33** | >32 | >32 | 32 | >32 | **2** | **2** | >32 | >32 | 32 | >32 | **8** | **8** | >32 | >32 |
|  | 2 | >32 | >32 | >32 | >32 | **0,68** | **0,50** | >32 | >32 | >32 | >32 | **2** | 2 | >32 | >32 | >32 | >32 | **8** | **8** | >32 | >32 |
| Hill_SB_C1875 | 1 | >32 | >32 | >32 | >32 | **4** | **2,11** | >32 | >32 | >32 | >32 | **8** | **8** | >32 | >32 | >32 | >32 | **8** | >32 | >32 | >32 |
|  | 2 | >32 | >32 | >32 | >32 | **1** | **2,16** | >32 | >32 | >32 | >32 | **2** | **8** | >32 | >32 | >32 | >32 | **8** | **8** | >32 | >32 |
