# Supplementary material for: Antimicrobial effects of novel Hermetia illucens peptides
Source: Sci Rep. 2026 Feb 24;16:10398. doi: 10.1038/s41598-026-40997-3 (PMC13031288; doi:10.1038/s41598-026-40997-3)
Supplement: Supplementary file 3 — Supplementary Material 3 [file 41598_2026_40997_MOESM3_ESM.docx]

**Supplementary Table 3** Comparative analysis of BSF-derived AMPs based on *in silico* prediction scores and experimental activity across multiple studies, including Moretta et al., Van Moll et al., and the present work.

| **Peptide Name** | ***In silico* Result/Activity (CAMP database)** | **Moretta et al.** | **Van Moll et al.** | **This Study** |
| --- | --- | --- | --- | --- |
| **Hill_BB_C46948** | SVM: 0.968  RF: 0.9375  ANN: AMP  DA: 0.999 | E. coli **ATCC 8739**: 3, 12 µM (120h) (No MIC) | P. aeruginosa **ATCC 9027**: 22.63, >32.00 µM  E. coli **ATCC 8739**: 1-3.57 µM | P. aeruginosa **ATCC 9027**: 0.94-1.46 µM |
| **Hill_LB_C16634** | SVM: 0.992  RF: 0.913  ANN: AMP  DA: 0.999 | E. coli **ATCC 8739**: 3, 12 µM (120h) (No MIC) | No Activity | No Activity |
| **NHill_AD_C53857** | SVM: 0.811  RF: 0.742  ANN: AMP  DA: 0.973 | No Activity | No Activity | No Activity |
| **NHill_AD_C49215** | SVM: 0.961  RF: 0.8735  ANN: AMP  DA: 0.999 | No Activity | No Activity | No Activity |
| **Hill_BB_C6571** | SVM: 0.950  RF: 0.9815  ANN: AMP  DA: 0.992 | E. coli **ATCC 8739**: 3, 12 µM (120h) (No MIC) | No Activity | No Activity |
| **Hill_BB_C7176** | SVM: 0.933  RF: 0.938  ANN: AMP  DA: 0.996 | - | S. aureus **ATCC 6538**: 0.50, 0.70 µM  P. aeruginosa **ATCC 9027**: 32->32 µM | ***E. coli* ATCC 8739: 1-4 µM**  P. aeruginosa: 11.37-16 µM  S. aureus **ATCC 6538**: 1.37-2.86 µM  B. cereus: 4-4.03 µM |
| **Hill_BB_C1827** | SVM: 0.898  RF: 0.8805  ANN: AMP  DA: 0.995 | - | - | E. coli **ATCC 8739**: 0.82-4 µM |
| **Hill_BB_C7985** | SVM: 0.991  RF: 0.9175  ANN: AMP  DA: 0.999 | E. coli **ATCC 8739**: 3, 12 µM (120h) (No MIC) | - | E. coli **ATCC 8739**: 3.10-3.97 µM |
| **Hill_BB_C3195** | SVM: 0.868  RF: 0.9945  ANN: AMP  DA: 0.988 | - | E. coli **ATCC 8739**: 0.32, 0.38 µM  P. aeruginosa **ATCC 9027**: 0.40, 0.78 µM | E. coli **ATCC 8739**: 1, 0.68 µM  P. aeruginosa **ATCC 9027**: 0.33, 0.50 µM |
| **Hill_SB_C1875** | SVM: 0.714  RF: 0.9115  ANN: AMP  DA: 0.949 | - | - | E. coli **ATCC 8739**: 1-4 µM  P. aeruginosa **ATCC 9027**: 2.11-2.16 µM |
